# The novel roles of virus infection-associated gene CDKN1A in chemoresistance and immune infiltration of glioblastoma

**DOI:** 10.18632/aging.202519

**Published:** 2021-02-17

**Authors:** Kuan Hu, Juanni Li, Geting Wu, Lei Zhou, Xiang Wang, Yuanliang Yan, Zhijie Xu

**Affiliations:** 1 Department of Hepatobiliary Surgery, Xiangya Hospital, Central South University, Changsha 410008, Hunan, China; 2 Department of Pathology, Xiangya Hospital, Central South University, Changsha 410008, Hunan, China; 3 Department of Anesthesiology, Third Xiangya Hospital of Central South University, Changsha 410008, Hunan, China; 4 Department of Pharmacy, National Clinical Research Center for Geriatric Disorders, Xiangya Hospital, Central South University, Changsha 410008, Hunan, China; 5 National Clinical Research Center for Geriatric Disorders, Xiangya Hospital, Central South University, Changsha 410008, Hunan, China

**Keywords:** chemoresistance, TMZ, CDKN1A, GBM, TILs

## Abstract

Chemoresistance is a common limitation for successful treatment of glioblastoma multiforme (GBM). Recently, virus infections have been demonstrated to be associated with tumorigenesis and chemoresistance in tumors. However, the role of infection-related genes in GBM haven’t been clearly demonstrated. Here, we explored the roles and mechanisms of human T-lymphotropic virus type-1 (HTLV-1) infections in tumorigenesis and chemoresistance in GBM. Four candidate genes, CDKN1A, MSX1, MYC and CHEK2, were identified to be the codifferentially expressed genes between three temozolomide (TMZ) chemotherapy datasets and one HTLV-1 infection gene set. Next, comprehensive bioinformatics data from several databases indicated that only CDKN1A was significantly upregulated in both GBM tissues and cells and showed the greatest prognostic value in GBM patients. Clinical data identified the correlations between CDKN1A expression and clinicopathological parameters of GBM patients. Moreover, CDKN1A was found to be involved in AKT-mediated TMZ resistance of glioma cells. In addition, KEGG analysis of CDKN1A-associated coexpression genes showed that CDKN1A was potentially involved in complement and coagulation cascades pathways in GBM. Finally, TISIDB database was used to investigate the role of CDKN1A in tumor-immune system interactions in GBM. These findings enhanced our understanding of the roles of CDKN1A in tumorigenesis and therapy response in GBM.

## INTRODUCTION

Glioma is the most common primary intracranial tumor and represents approximately 50% of primary brain tumors that cause significant mortality and morbidity [1–3]. Despite very intensive treatments, glioblastoma multiforme (GBM), which is the most aggressive and devastating glioma histology, has a median overall survival of less than 2 years [4]. Surgery, chemotherapy and radiotherapy are the conventional treatments for GBM, and chemoresistance is a common limitation of successful GBM treatment [5, 6]. Recently, studies have indicated that virus infection-associated chemoresistance has been reported in several cancers [7, 8] but their role in GBM treatment has not been clearly demonstrated. Hence, exploring the roles and mechanisms of virus infection-associated chemoresistance in GBM is a critical need and may offer promise for a novel therapeutic biomarker in GBM.

Cyclin dependent kinase inhibitor 1A (CDKN1A), a cell cycle inhibitor, is directly controlled by p53-dependent or independent pathways and is involved in terminal differentiation, stem cell renewal, apoptosis and cell migration. It has become increasingly clear that CDKN1A can function as an oncogene or as a classic tumor suppressor [9]. Serving as an oncogene, cytoplasmic CDKN1A was reported to promote oncogenic transformation of HER-2-positive breast cancer cells [10]. In contrast, CDKN1A inhibition by LincRNAFEZF1-AS1 promoted cell proliferation in gastric cancer [11]. Moreover, CDKN1A was also reported to play a critical role in the immune microenvironment in tumors. Price JG et al. demonstrated that CDKN1A can regulate Langerhans cell survival and promote Treg cell generation upon exposure to ionizing radiation in cutaneous tumors [12]. However, the detailed function and mechanism of CDKN1A in the tumorigenesis and immune infiltration of GBM have not yet been investigated.

Several studies have reported that some key factors related to the immune response were obviously altered in GBM and subsequently resulted in tumor immune evasion [13–15]. In addition to the classic treatments (e.g. surgery, chemotherapy and radiotherapy), immune therapy is increasingly considered a particularly promising treatment modality for tumors that stimulates the immune system and activates specific immune cells to attack tumor cells [16, 17]. Several parameters related to the immune system have been identified to predict prognoses for some glioma histologies [18, 19]. However, comprehensive research on the immune microenvironment of GBM is still rare and needs further exploration.

The purpose of the present study was to investigate the detailed roles and mechanisms of human T-lymphotropic virus type-1 (HTLV-1) infection-related genes for GBM chemotherapy. By analyzing the available data from several public databases, the HTLV-1 infection-related gene, CDKN1A, was found to influence the GBM chemotherapy response. Moreover, higher expression of CDKN1A was identified in GBM tissues and cell lines and was correlated with poor prognoses of GBM patients. Furthermore, by colony formation assay, transient transfection assay and western blot, we found that CDKN1A was highly expressed in TMZ-resistant glioma cells and involved in AKT-mediated TMZ resistance of glioma cells.

## RESULTS

### Identification of differentially expressed genes between the cases and controls

**Figure 1:**
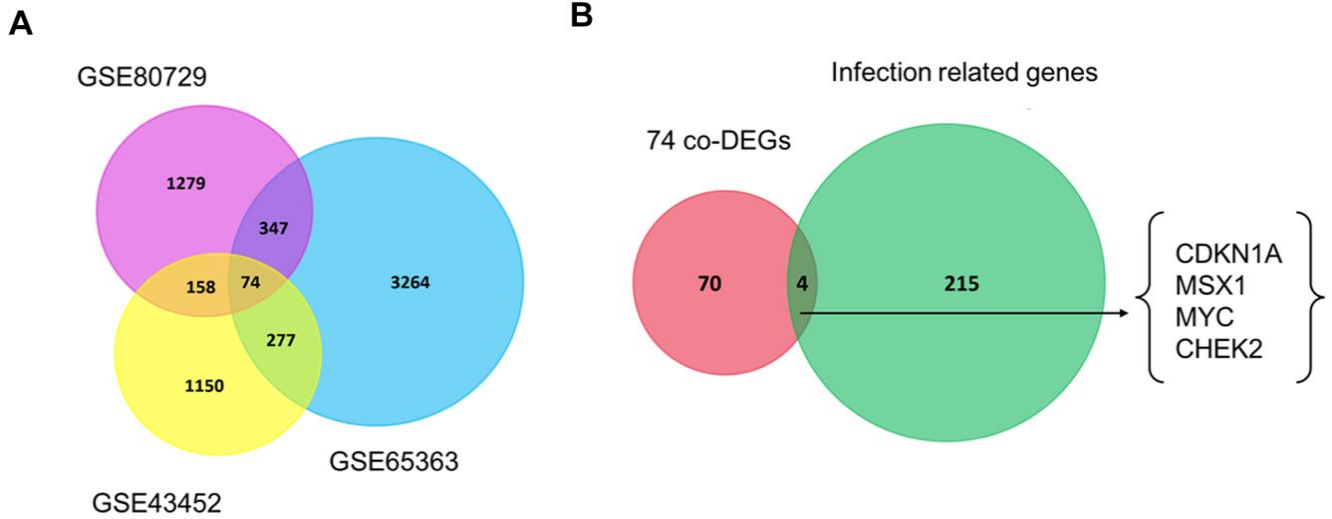
**Venn diagrams of gene expression microarray datasets.** (**A**) The 74 co-DEGs in three publicly available datasets including GSE43452, GSE65363 and GSE80729. (**B**) CDKN1A, MSX1, MYC and CHEK2 were consistently identified between 74 co-DEGs and one HTLV-1 infection-related gene set. Each rectangle represents a dataset. The number in each overlapping region represents the number of differentially expressed genes. The intersection in the middlemost area represents the number of genes that were consistently differentially expressed in all these datasets.

Recently, the development of resistance to TMZ has become a common limitation for successful GBM treatment [20]. To screen the co-DEGs between the untreated group and TMZ-treated group, we analyzed the gene expression profiles from three publicly available datasets regarding TMZ chemotherapy from the GEO platform, namely, GSE43452, GSE65363 and GSE80729. Using the screening criteria of p-value < 0.05, we identified 1659 genes in GSE43452, 3962 genes in GSE65363 and 1858 genes in GSE80729 (Table 1). Next, using the Venn analysis provided by FunRich, 74 genes were found to be significantly codifferentially expressed in all three datasets (Figure 1A, Supplementary Table 1). The co-DEGs from three datasets were presumed to have an impact on the chemotherapy response of GBM.

**Table 1:** The main characteristics of 3 selected studies on gene expression profiling by microarray.

**GEOa datasets**	**Platform**	**Samples size**		**DEGsb**	**Co-DEGs**	**Submission** **date**	**References**
		**Control**	**Treatment**				
GSE43452	GPL10558	2	2	1659		Jan 11, 2013	[46]
GSE65363	GPL570	3	3	3962	74	Jan 28, 2015	N/A c
GSE80729	GPL10558	3	3	1858		Apr 27, 2016	[47]

Note: ^a^GEO: Gene Expression Omnibus datasets.

^b^DEGs: differentially expressed genes.

^c^Further information can be found at https://www.ncbi.nlm.nih.gov/geo/.

Virus infections have been reported to be involved in the development of GBM [21, 22]. Next, we explored the roles of HTLV-1 infections on the development and chemoresistance of GBM. The HTLV-1 infection-related gene set was extracted from the MalaCards database and four HTLV-1 infection-related genes, namely, CDKN1A, MSX1, MYC and CHEK2, were identified in the aforementioned co-DEGs of the three datasets (Figure 1B). These four genes were hypothesized to have an influence on virus infection-associated chemoresistance in GBM.

### CDKN1A shows the greatest prognostic value in GBM

**Figure 2:**
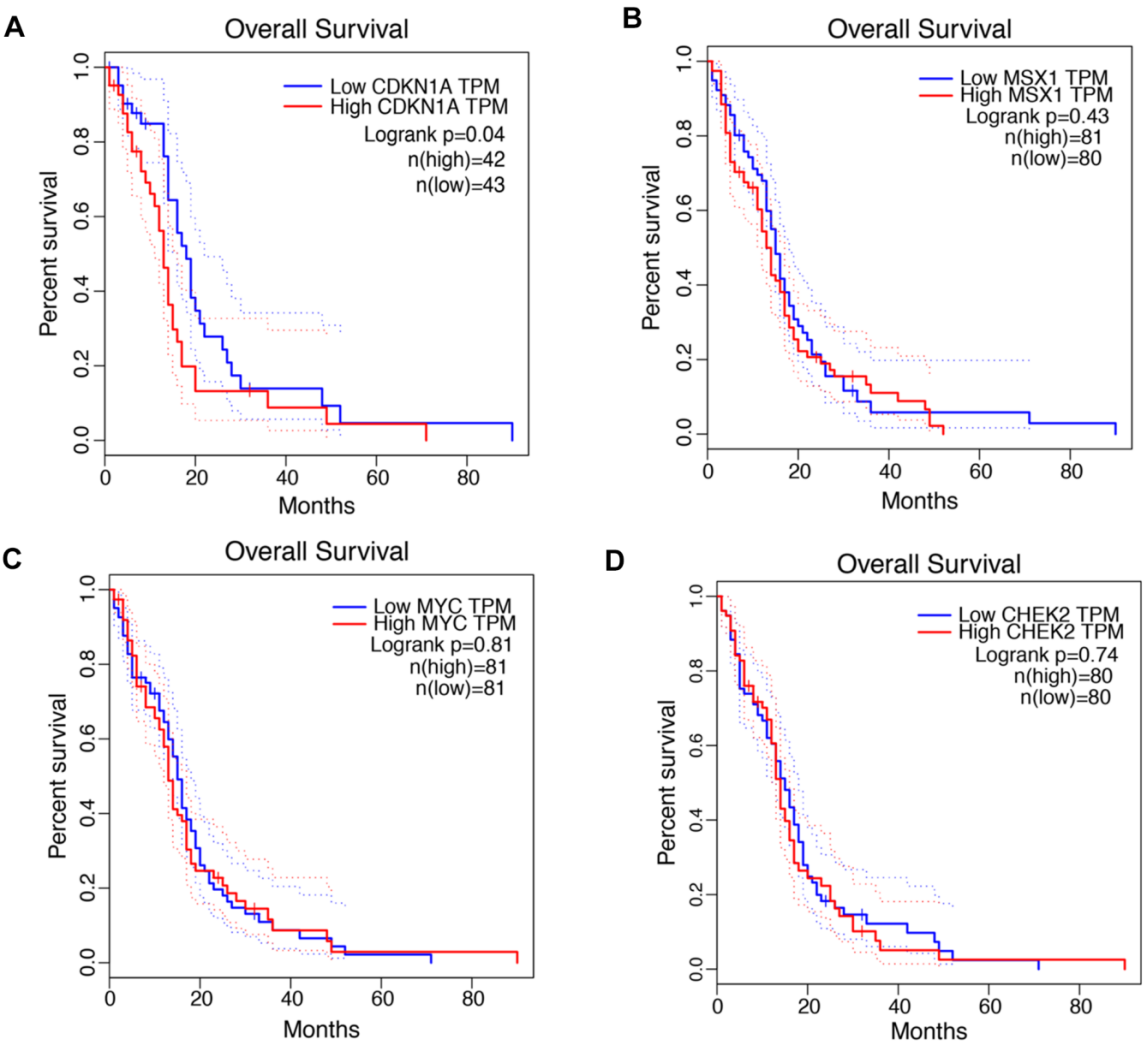
**Prognostic values of CDKN1A, MSX1, MYC and CHEK2 in GBM.** (**A**–**D**) Kaplan-Meier analysis of overall survival between the samples with high expression of the four genes and those with low expression in GBM by using the GEPIA database.

The associations between the expression levels of CDKN1A, MSX1, MYC and CHEK2 and prognosis in GBM patients were analyzed using the GEPIA database. Notably, high CDKN1A expression was marginally associated with poor prognosis in GBM patients (Figure 2A), but the other three genes did not exhibit significant prognostic value (Figure 2B, 2C). Similar findings were identified by the GlioVis database. As shown in Supplementary Figure 1, CDKN1A expression levels significantly impacted prognosis in GBM but MSX1, MYC and CHEK did not have obvious associations with survival in GBM. Based on these results, CDKN1A, as the only gene that showed obvious prognostic significance in GBM, was selected for further study.

We then analyzed the correlation between CDKN1A expression levels and clinicopathological parameters of GBM patients. The clinical data of GBM patients were downloaded from the Wanderer database. As shown in Table 2, CDKN1A expression was significantly associated with age (p = 0.030) and vital status (p = 0.011).

**Table 2:** Correlation of CDKN1A with clinicopathological parameters in GBM.

**Characteristics**	**Case**	**CDKN1A**	
		**Mean ± SD**	**P-value**
**Gender**			
Female	20	11.35 ± 1.27	
Male	26	11.20 ± 0.94	0.451^b^
**Age**			
≥ 50	39	11.43 ± 0.92	
< 50	7	10.34 ± 1.51	0.030^b^
**Race**			
White	44	11.26 ± 1.03	
Black or African American	2	11.40 ± 2.60	0.829^b^
**Ethnicity**			
Not Hispanic or Latino	30	11.20 ± 1.15	
Hispanic or Latino	1	11.30 ± 0.00	0.955^b^
**Vital status**			
Alive	22	10.78 ± 1.11	
Dead	24	11.71 ± 0.86	0.011^b^
**Tumor status**			
With tumor	36	11.30 ± 1.18	
Tumor free	5	11.28 ± 0.68	0.858^b^
**Performance status timing**			
Pre-adjuvant therapy	20	11.29 ± 1.20	
Post-adjuvant therapy	2	10.52 ± 2.19	
Pre-operative	5	11.39 ± 0.52	0.828^a^

Significant P values are underlined.

^a^Kruskal–Wallis rank test.

^b^Mann–Whitney U test.

### CDKN1A is upregulated in GBM tissues and cell lines and impacts the treatment outcomes of GBM patients

**Figure 3:**
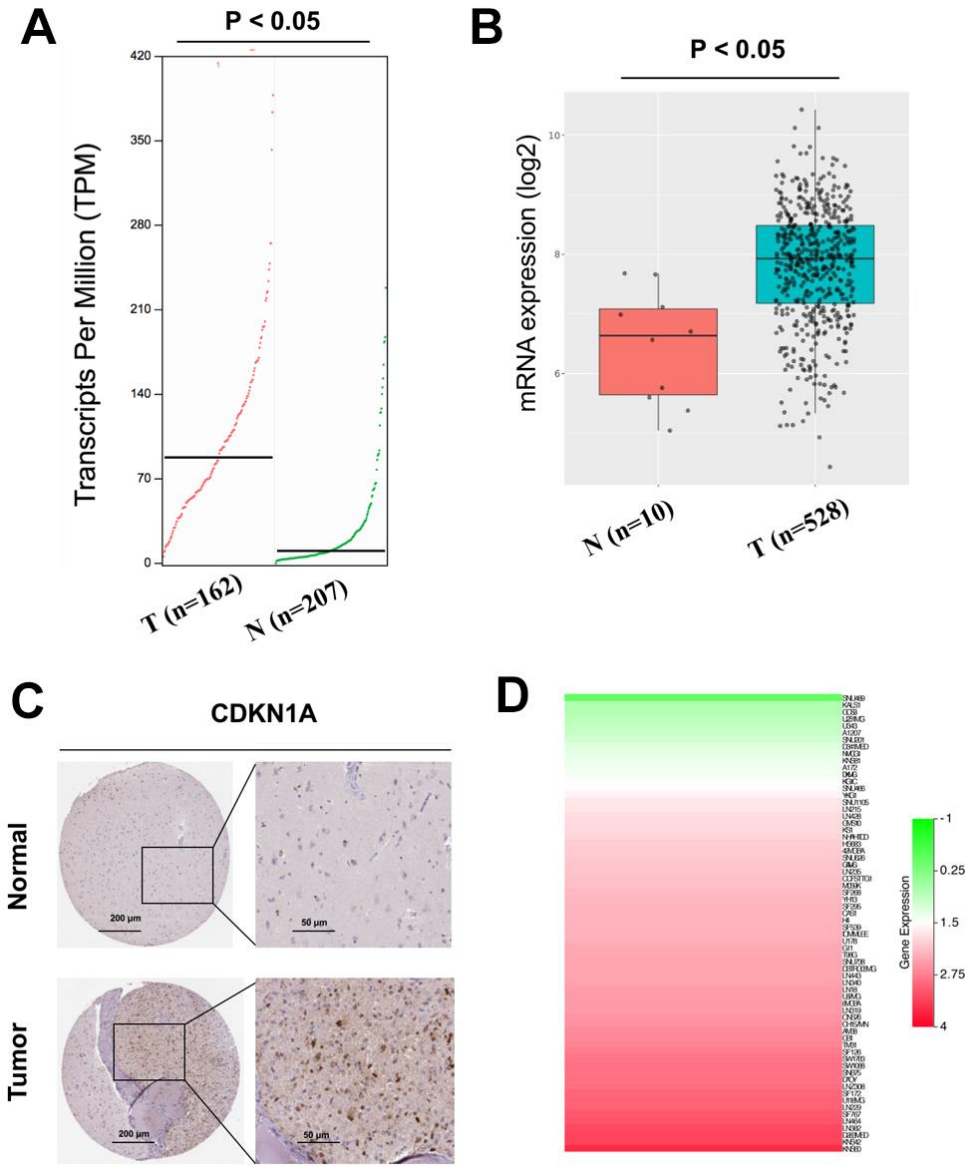
**Analysis of the CDKN1A expression levels in GBM tissues and cell lines.** (**A**, **B**) The mRNA expression of CDKN1A in GBM tissues was detected by using the GEPIA and GlioVis databases. (**C**) the Human Protein Atlas project showed representative immunohistochemical images of CDKN1A in GBM tissues compared with surrounding normal tissues. (**D**) The expression levels of CDKN1A in GBM cell lines was detected by using the CCLE database.

The expression profiles of CDKN1A were analyzed using two independent bioinformatics databases, GEPIA and GlioVis. First, the analysis results from the GEPIA database indicated that CDKN1A mRNA expression was higher in GBM tissues than that in noncancerous tissues (Figure 3A). Next, data from the GlioVis database revealed that CDKN1A mRNA expression levels were significantly upregulated in GBM tissues (Figure 3B), which was consistent with those obtained using the GEPIA database. Both databases demonstrated that CDKN1A mRNA expression levels were upregulated in GBM tissues. Meanwhile, we evaluated the protein expression levels of CDKN1A in GBM patients by analyzing the immunohistochemical data from the Human Protein Atlas. As shown in Figure 3C, significantly elevated levels of CDKN1A was found in the glioma tissues. Further analysis using the CCLE database to study CDKN1A expression profiles in GBM cell lines and the heatmap revealed elevated CDKN1A expression levels in most GBM cell lines (Figure 3D).

**Figure 4:**
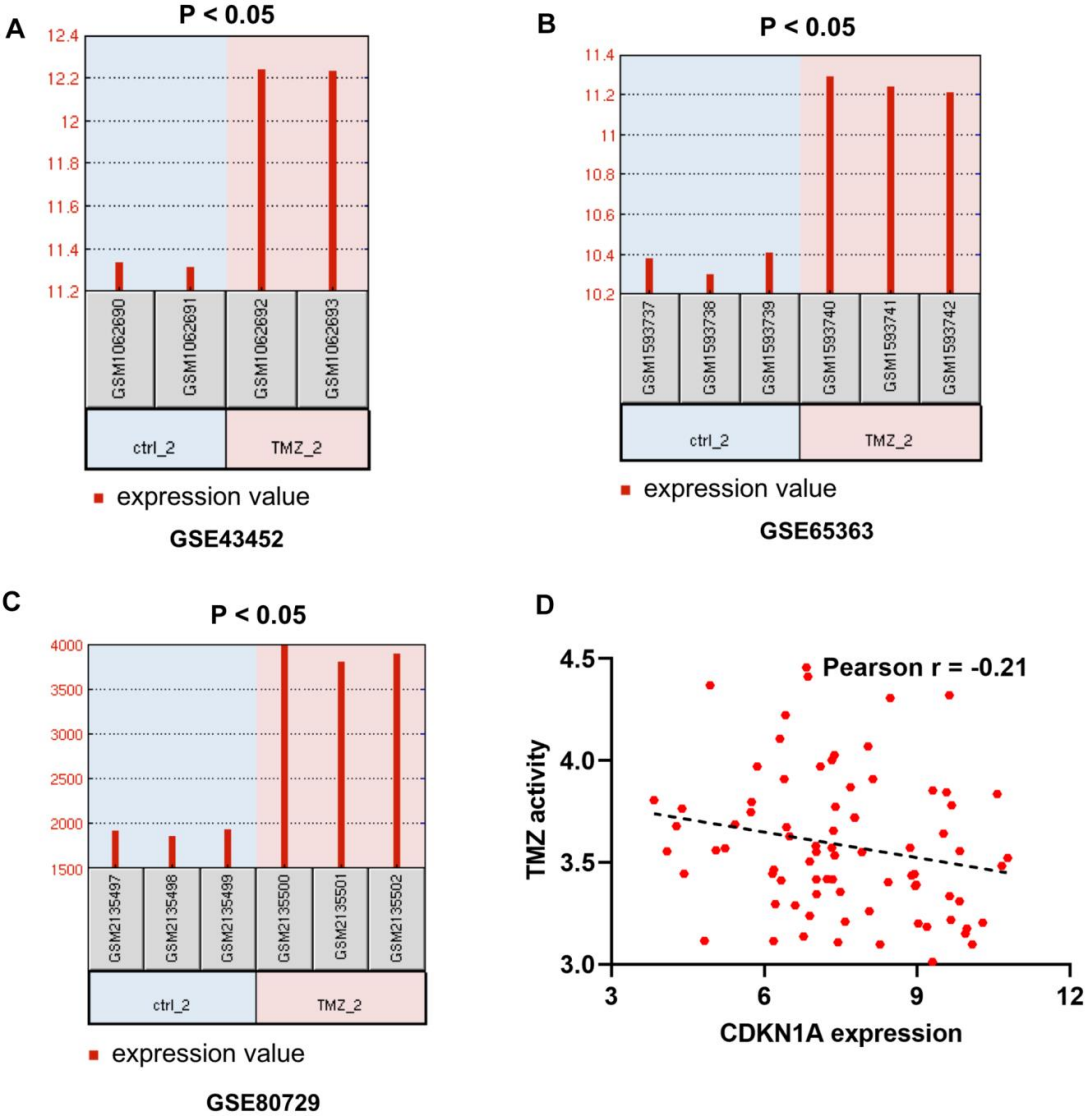
**The effect of CDKN1A on the therapeutic response of GBM patients.** (**A**–**C**) GSE43452, GSE65363 and GSE80729 are three microarray datasets related to TMZ chemotherapy and are employed to identify the impacts of CDKN1A expression levels on GBM therapy. (**D**) the expression levels of CDKN1A might be negative correlation with TMZ activity in 79 glioma cells from CellMinerCDB.

To further determine the effect of CDKN1A on the treatment outcomes of GBM patients, we checked the expression level of CDKN1A in three microarray datasets related to TMZ chemotherapy. The data from these three datasets, GSE43452, GSE65363 and GSE80729, all showed that treatment with the anticancer agent TMZ clearly upregulated CDKN1A expression in human GBM cells (Figure 4A–4C). In addition, the expression levels of CDKN1A might be negative correlation with TMZ activity in 79 glioma cells from CellMinerCDB [23] (Figure 4D, Supplementary Table 4).

The above results indicated that CDKN1A expression levels were upregulated in both GBM tissues and cell lines and may influence the chemotherapy responses of GBM patients.

### CDKN1A was involved in TMZ resistance of glioma cells

**Figure 5:**
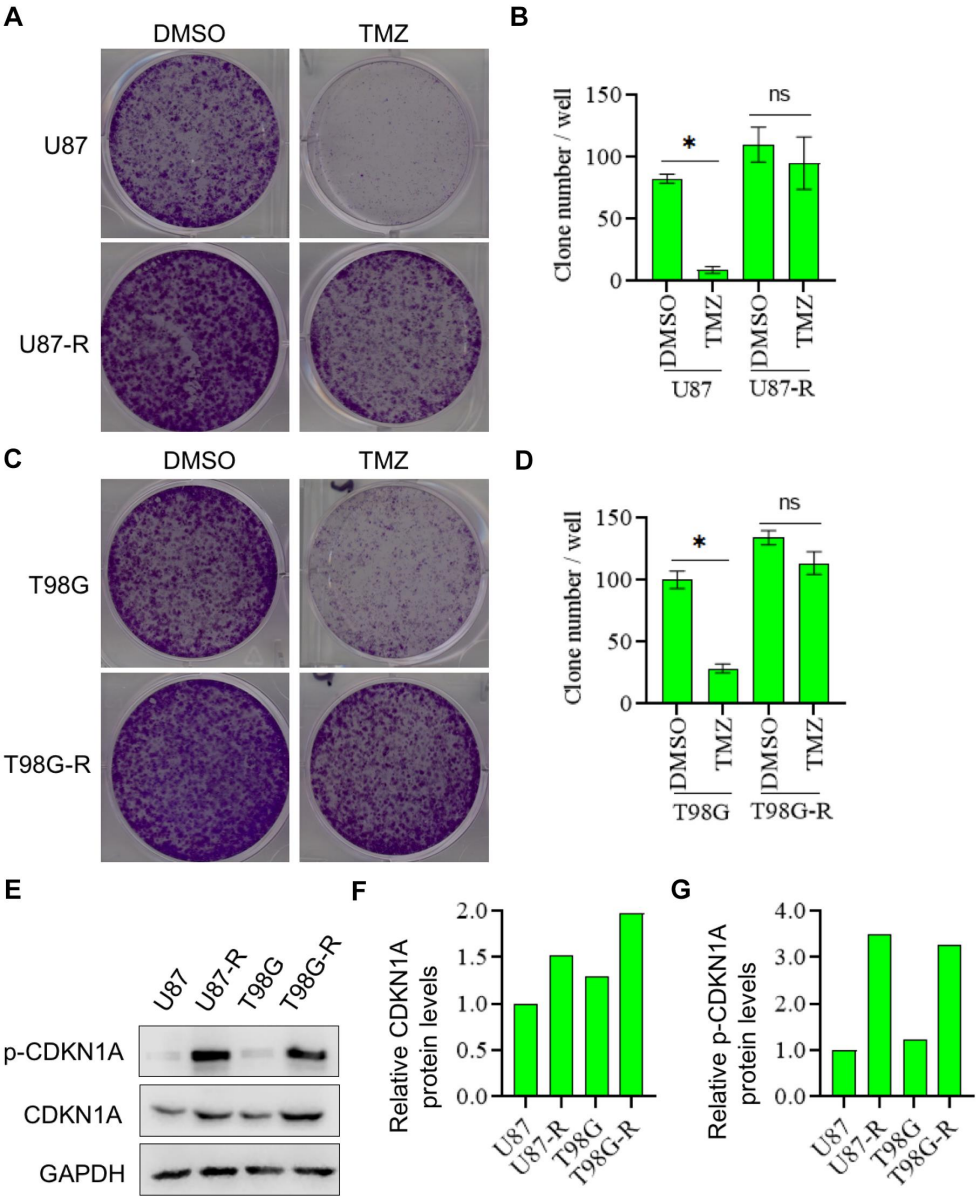
**CDKN1A and p-CDKN1A were highly expressed in TMZ-resistant glioma cells.** (**A**, **B**) Compared with U87 cells, U87-R cells were significantly more resistant to the therapy of TMZ by using the colony formation assay. (**C**, **D**) Compared with T98G cells, T98G-R cells were significantly more resistant to the therapy of TMZ by using the colony formation assay. (**E**–**G**) Compared with U87 and T98G cells, the protein expression levels of CDKN1A and p-CDKN1A were significantly higher in U87-R and T98G-R cells, respectively. The results were presented as means ± SD (n = 3 for each panel). Statistical significance was concluded at *P < 0.05.

To verify the promoting effect of CDKN1A on cell chemoresistance, we detect CDKN1A expression in TMZ-resistant glioma cell lines (U87-R and T98G-R cells) and their parental cell lines (U87 and T98G cells). First, using the colony formation assay, we proved that U87-R and T98G-R cells were indeed significantly more resistant to the therapy of TMZ compared with U87 and T98G cells, respectively (Figure 5A–5D). After this, CDKN1A and p-CDKN1A expression was further found to be higher in U87-R and T98G-R cells compared with that in U87 and T98G cells, respectively (Figure 5E–5G). These data thus suggested that CDKN1A may played a critical role in TMZ resistance of GBM cells.

**Figure 6:**
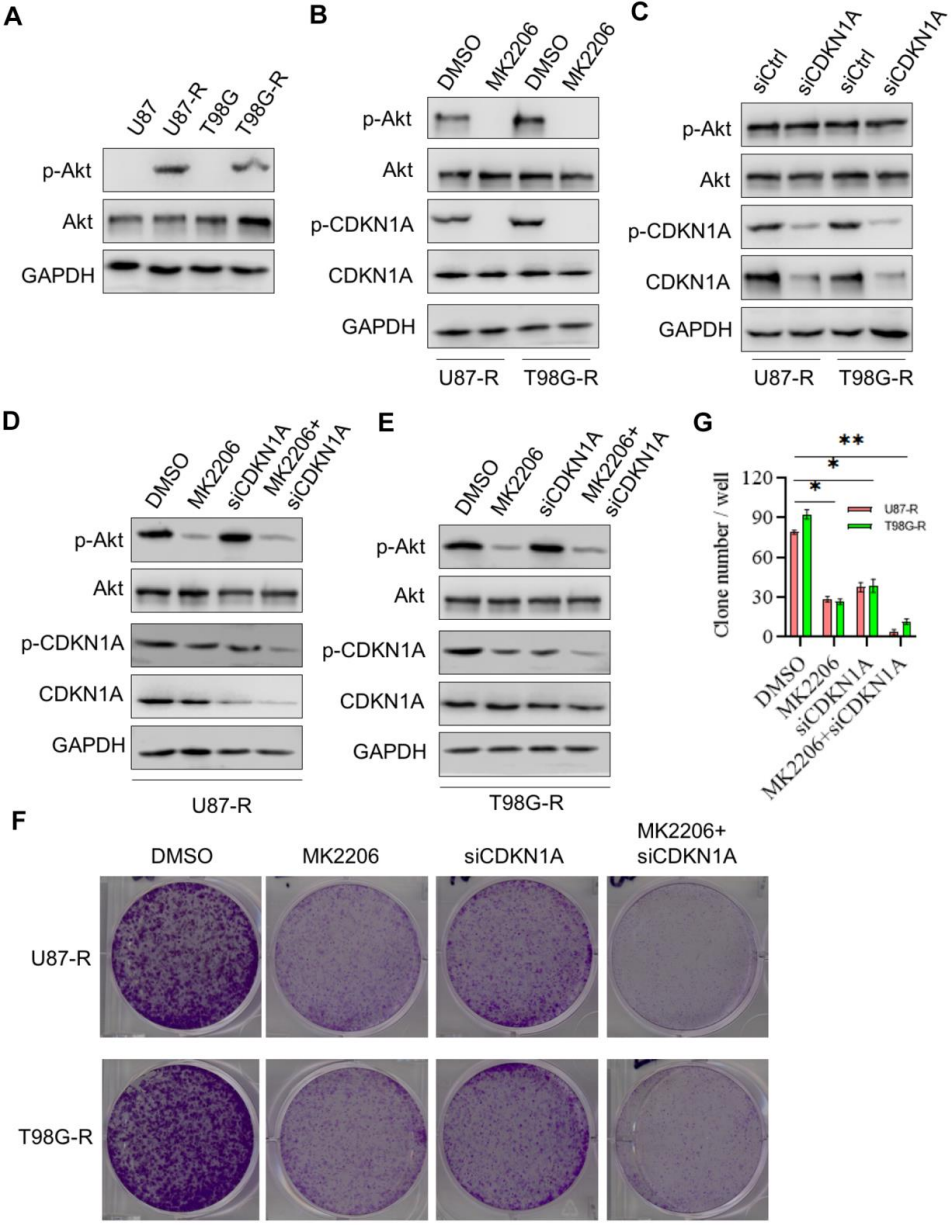
**CDKN1A was involved in TMZ resistance of glioma cells.** (**A**) Western blot for phospho-Akt (Ser-473), Akt in U87, U87-R T98G, and T98G-R cells. (**B**) Western blot for phospho-Akt (Ser-473), Akt, phospho-CDKN1A, and CDKN1A in U87-R or T98G-R cells treated with DMSO and the specific inhibitor to AKT MK2206. (**C**) Western blot for phospho-Akt (Ser-473), Akt, phospho-CDKN1A, and CDKN1A in U87-R or T98G-R cells treated with siCtrl and siCDKN1A. (**D**) Western blot for phospho-Akt (Ser-473), Akt, phospho-CDKN1A, and CDKN1A in U87-R cells treated with DMSO, MK2206, siCDKN1A and MK2206 plus siCDKN1A. (**E**) Western blot for phospho-Akt (Ser-473), Akt, phospho-CDKN1A, and CDKN1A in T98G-R cells treated with DMSO, MK2206, siCDKN1A and MK2206 plus siCDKN1A. (**F**, **G**) Colony formation assay of U87-R or T98G-R cells treated with DMSO, MK2206, siCDKN1A and MK2206 plus siCDKN1A. The results were presented as means ± SD (n = 3 for each panel). Statistical significance was concluded at *P < 0.05, **P < 0.01.

Studies have shown that AKT signaling pathway plays an important role in chemoresistance of GBM [24]. Thus, we then investigated whether the effect of CDKN1A on cell chemoresistance was associated with AKT activity. Western blot analysis showed that the expression of p-AKT, a constitutively active form of AKT, was significantly higher in TMZ-resistant glioma cells compared with that in their parental cells, whereas the expression of total AKT has no change, indicating that AKT played a critical role in chemoresistance of GBM (Figure 6A). We then treated TMZ-resistant glioma cells with the specific inhibitor to AKT, MK2206, and found that MK2206 treatment significantly suppressed phosphorylation of CDKN1A at Ser473 (Figure 6B). However, the down-regulated expression of CDKN1A had no effect on AKT expression in TMZ-resistant glioma cells, suggesting that CDKN1A was a downstream regulatory molecule of AKT (Figure 6C). Next, we treated TMZ-resistant glioma cells with both MK2206 and siCDKN1A, as shown in Figure 6D, 6E, the inhibitory effect on p-CDKN1A expression was remarkably increased by combinational treatment. We performed a colony formation assay and observed that TMZ-resistant glioma cells treated with both MK2206 and siCDKN1A had a significantly lower number of colony formation compared with the control group and either of the individual treatment groups (Figure 6F, 6G). The above results indicated that CDKN1A was involved in AKT-mediated TMZ resistance of glioma cells.

### Functional enrichment analysis of CDKN1A-associated coexpression genes

**Figure 7:**
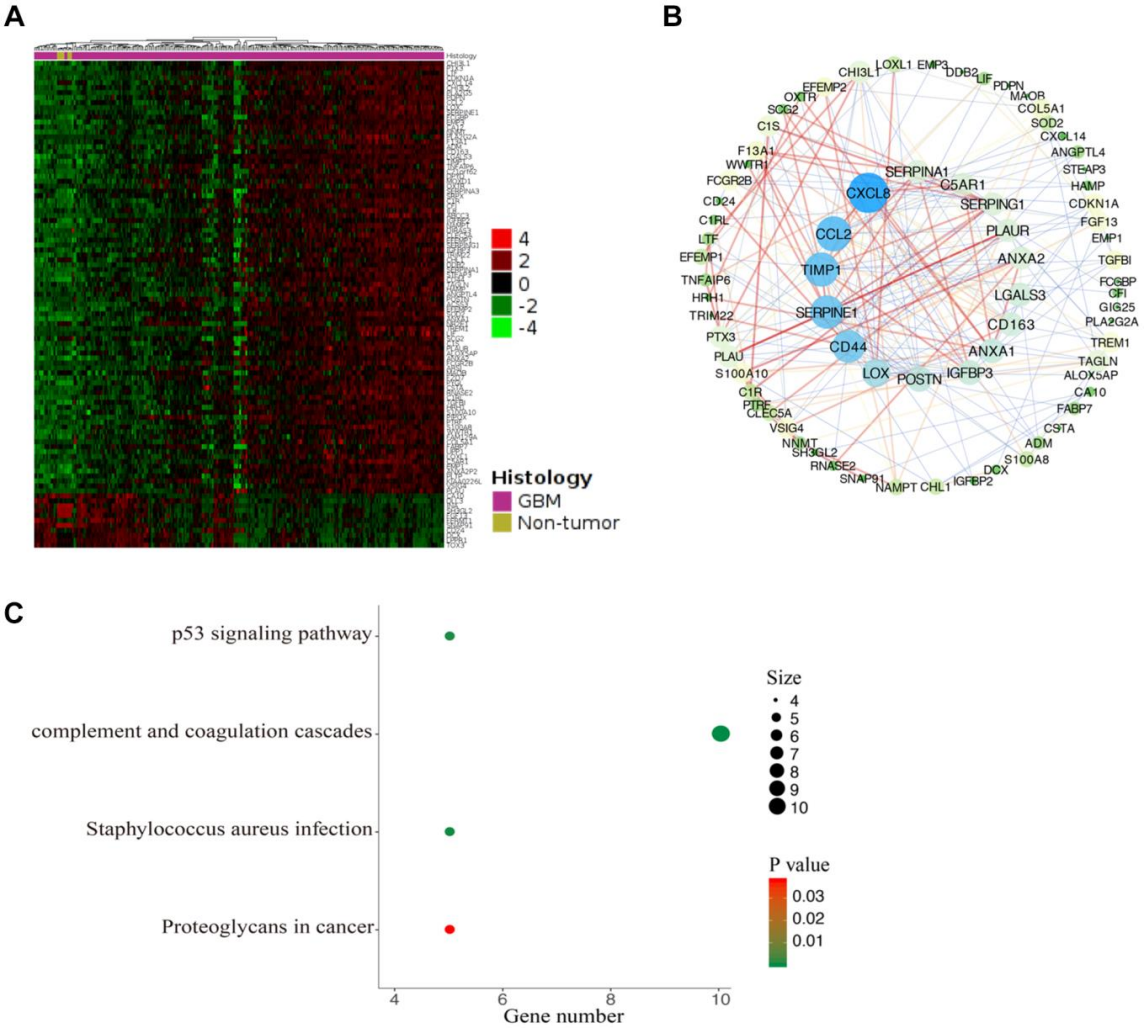
**Functional enrichment analysis of CDKN1A-associated coexpression genes.** (**A**) The coexpression genes of CDKN1A were shown as a heatmap via GlioVis. (**B**) The PPI network of CDKN1A-associated coexpression genes was created by the STRING and Cytoscape software. (**C**) The significant KEGG pathways associated with the CDKN1A coexpression genes were identified using the DAVID database.

To further understand the potential role of CDKN1A in GBM development, we performed functional enrichment annotation analysis of its coexpressed genes. We extracted the datasets (TCGA-GBM) from TCGA to screen DEGs that interact with CDKN1A from the GlioVis database. As shown in Figure 7A and Supplementary Table 3, 99 genes were acquired using screening criteria of |LogFC| ≥1.3 and P≤0.05. Then, a PPI network of the genes that coexpressed with CDKN1A was created with the STRING database and Cytoscape software (Figure 7B). To further understand the biological functions of these coexpressed genes, KEGG pathway analyses were conducted by using the DAVID database. The results showed that these coexpressed genes were functionally enriched in several pathways which were mostly those involving complement and coagulation cascades (Figure 7C).

### Regulation of immune molecules by CDKN1A

**Figure 8:**
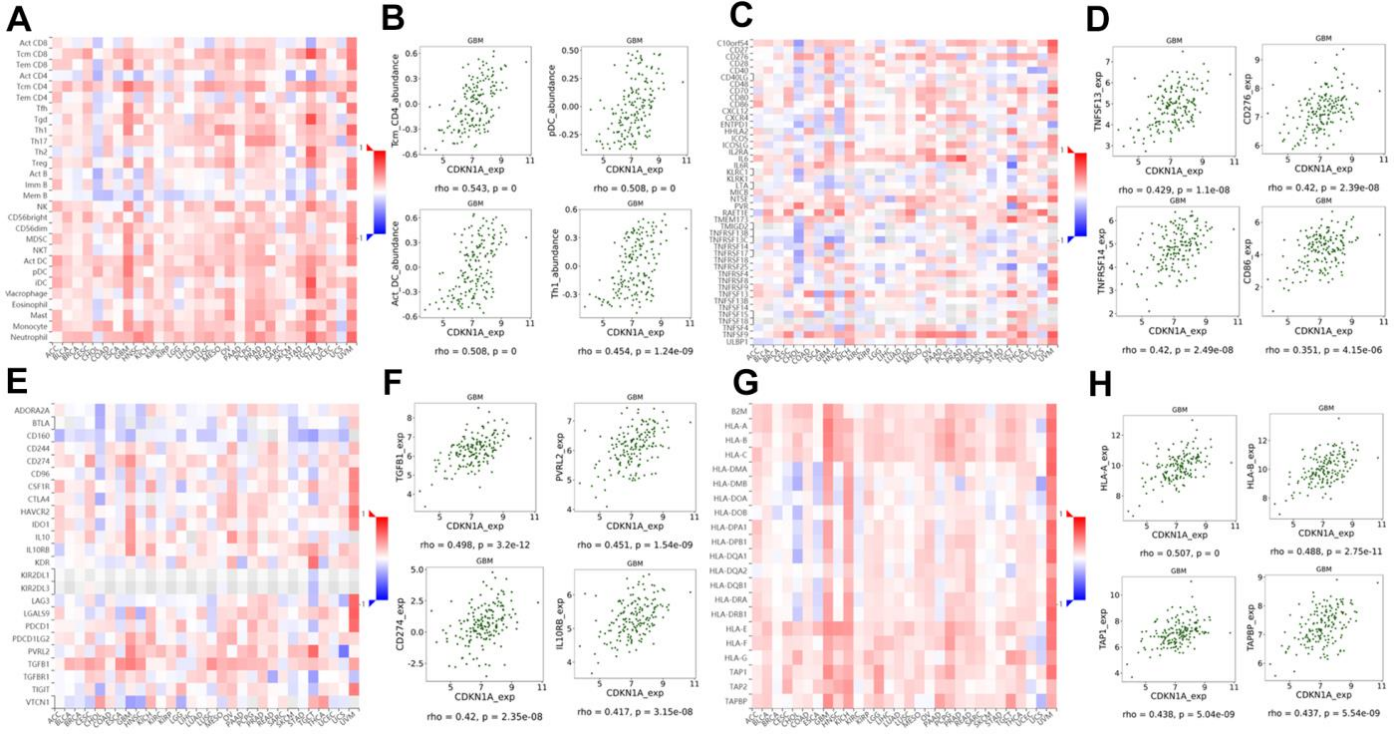
**Correlation of CDKN1A expression with lymphocytes and immunomodulators in GBM.** (**A**) The correlation between CDKN1A expression and TILs. (**B**) The top four TILs showing the most significant correlations with CDKN1A expression. (**C**) The correlation between CDKN1A expression and immunostimulators. (**D**) The top four immunostimulators showing the most significant correlations with CDKN1A expression. (**E**) The correlation between CDKN1A expression and immunoinhibitors. (**F**) The top four immunoinhibitors showing the most significant correlation with CDKN1A expression. (**G**) The correlation between CDKN1A expression and MHC molecules. (**H**) The top four MHC molecules showing the most significant correlation with CDKN1A expression.

Guglietta S et al. reported that the complement and coagulation cascade pathways were associated with protumorigenic phenotypes of immune cells and protection of tumor cells from immune attack which ultimately favor the development and metastasis of tumors [25]. Therefore, we investigated the associations between CDKN1A expression and lymphocytes and immunomodulators using the TISIDB database. Figure 8A shows the positive correlation between CDKN1A expression and tumor-infiltrating lymphocytes (TILs) in GBM patients. Additionally, the lymphocytes that exhibited the most significant correlations included central memory CD4 T cells (Tcm_CD4, Spearman: ρ= 0.543), plasmacytoid dendritic cells (pDC, Spearman: ρ= 0.508), activated dendritic cells (Act_DC, Spearman: ρ= 0.508) and type-1 T helper cells (Th1, Spearman: ρ= 0.454) (Figure 8B). Immunomodulators have been classified into three types of molecules which include immunoinhibitors, immunostimulators and major histocompatibility complex (MHC) molecules. Figure 8C shows the correlation between CDKN1A expression and immunostimulators; the immunostimulators displaying the most significant correlations included TNFSF13 (Spearman: ρ= 0.429), CD276 (Spearman: ρ= 0.420), TNFRSF14 (Spearman: ρ= 0.420) and CD86 (Spearman: ρ= 0.351) (Figure 8D). Figure 8E shows the correlations between CDKN1A expression and immunoinhibitors; those immunoinhibitors displaying the most significant correlations included TGFB1 (Spearman: ρ= 0.498), PVRL2 (Spearman: ρ= 0.451), CD274 (Spearman: ρ= 0.420) and IL10RB (Spearman: ρ= 0.417) (Figure 8F). Figure 8G shows the correlations between CDKN1A expression and MHC molecules; the MHC molecules that showed the most significant correlations included HLA-A (Spearman: ρ= 0.507), HLA-B (Spearman: ρ= 0.488), TAP1 (Spearman: ρ= 0.438), and TAPBP (Spearman: ρ= 0.437) (Figure 8H). We speculate that since CDKN1A was significantly correlated with various types of tumor-infiltrating lymphocytes, immunoinhibitors, immunostimulators and MHC molecules in GBM, it might exert a more significant effect on immune fingerprinting in GBM.

## DISCUSSION

Our research group aimed to investigate critical and novel biomarkers involved in the development of virus infection-associated chemoresistance of GBM. Intriguingly, four genes including CDKN1A, MSX1, MYC and CHEK2 were identified by screening co-DEGs in three TMZ chemotherapy-related databases and one HTLV-1 infection-related gene set. However, only CDKN1A expression showed a significant correlation with poor prognosis in GBM patients with remarkable consistency between two independent bioinformatics platforms. CDKN1A, which was also found to be upregulated in GBM tissues and cell lines and impacted treatment outcomes of GBM patients, was selected for further in-depth investigations of its biological processes and signaling pathways as well as its correlations with immune regulation.

CDKN1A is a cyclin-dependent kinase inhibitor [26, 27], and an important versatile cell cycle regulator that is involved in cell migration and autophagy and is often deregulated in various cancers [9]. CDKN1A appears to exhibit dual-role behavior and acts either as an oncogene or tumor suppressor depending on the cell types or cellular localization [28]. A complex of cytoplasmic CDKN1A together with ectopic expressed cytosol-resided p53/CDKN1A complex suppressed cell invasion by targeting Bcl-2 family proteins in non-small cell lung cancer [29]. It has also been shown that the wild-type p53/CDKN1A complex can induce slug protein degradation and further suppress cell invasion [30]. In an opposite manner, Akt-activated CDKN1A was found to accelerate tumor onset and promote lung metastasis *in vivo* [31]. Cytoplasmic CDKN1A was reported to promote cell migration and invasion abilities in gastric cancer [32]. Similarly, Kreis NN et al. revealed that suppression of CDKN1A could inhibit migration and invasion in various cancer cell lines [33]. In some cases, CDKN1A phosphorylation by activated AKT1 prevents the nuclear translocation of CDKN1A and retains it in the cytoplasm, which is crucial for the pro-survival functions of CDKN1A [34, 35]. Cytoplasmic CDKN1A exhibits its oncogenic function might be dependent on non-traditional cytoplasmic targets of the protein. However, the detailed roles of CDKN1A in human brain tumors, especially GBM, have rarely been studied. In this study, we first demonstrated the prognostic value and potential roles of CDKN1A in GBM biology. We found that CDKN1A was involved in AKT-mediated TMZ resistance of glioma cells.

Recently, increasing evidence has indicated that tumor microenvironments and immune infiltration play a vital role on tumor development and chemoresistance [36–39]. Immunotherapy has emerged as a powerful tool for tumor treatment that targets and attacks tumor cells by stimulating the body's own immune system to recognize tumor cells and activate specific immune cells [40]. Glioblastoma is a very lethal form of human brain cancer with an extremely poor prognosis, which makes the development of novel therapeutic strategies targeting GBM of paramount importance. Immune therapy with the combination of conventional treatment (such as surgery, chemotherapy, and radiation) represents a particularly promising approach [41]. In this study, the correlation between CDKN1A and the immune system was assessed with the TISIDB database, and the results revealed that CDKN1A had the highest correlation with tumor-infiltrating lymphocytes including Tcm_CD4, pDC, Act_DC and Th1. Furthermore, CDKN1A had the most significant correlations with immunostimulators (such as TNFSF13, CD276, TNFRSF14 and CD86), immunoinhibitors (such as TGFB1, PVRL2, CD274 and IL10RB) and MHC molecules (such as HLA-A, HLA-B, TAP1 and TAPBP). DC-based vaccination is a basic form of immunotherapy and is critical for initiating and boosting anti-GBM immunity. Audencel, a DC-based cancer vaccine, was found to significantly upregulate Th1-related immunovariables and had effects on the immune system of GBM patients [42]. CD276 (B7-H3) is an immune checkpoint molecule and plays critical roles in T-cell suppression in GBM, which leads to better understanding of target pathways for immunotherapy in GBM [43]. As the most well-known recent biomarker in the cancer research field, PD-L1, also named CD274, was found to be upregulated in IDH1/2 wild-type GBM [44]. Blockade of the PD-L1/PD-1 axis has been reported to reduce Treg expansion and further improve T cell function and thus prevent immunosuppression in GBM [45]. Together, these findings suggest that CDKN1A, which is associated with these immune molecules, plays a vital role in immune escape in GBM microenvironments and can potentially serve as an immunotherapeutic target for GBM.

Nevertheless, there were several limitations in the present study that need to be mentioned. First, patient ethnicities in the TCGA database were mainly white and black, and more studies including other ethnic groups should be investigated further. Additionally, most of the datasets analyzed here were collected retrospectively, so further prospective studies are needed. Moreover, although attractive findings were identified in this study, more functional and mechanistic experiments and large-scale clinical trials are still required to confirm the clinical application value of CDKN1A.

In summary, this is the first report that CDKN1A is associated with the HTLV-1 infection-related chemoresistance of GBM and shows a significant correlation with poor prognosis. Moreover, CDKN1A is upregulated in GBM and might serve as a promising biomarker in the treatment of GBM patients. Furthermore, CDKN1A was found to be involved in AKT-mediated TMZ resistance of glioma cells. In addition, CDKN1A expression is related to tumor-infiltrating lymphocytes and immunomodulators. Therefore, our findings suggest that CDKN1A likely plays a critical role in immune cell infiltration and is a promising prognostic biomarker in patients with GBM.

## MATERIALS AND METHODS

### Data acquisition and reanalysis using different bioinformatics tools

The roles of HTLV-1 infection-associated chemoresistance of GBM were explored using several bioinformatics resources which are summarized in Table 3. Three temozolomide (TMZ) therapeutic transcriptome microarray datasets, GSE43452 [46], GSE65363 and GSE80729 [47], were downloaded from the gene expression omnibus (GEO) database (Table 1) [48]. Codifferentially expressed genes (co-DEGs) were identified in these three datasets using a Venn diagram. Next, we downloaded a comprehensive HTLV-1 infection related gene set from the MalaCards database (Supplementary Table 2) [49]. CDKN1A and other three HTLV-1 infection related genes were identified in the co-DEGs of the three GEO datasets and a corresponding Venn diagram was created by FunRich software [50].

**Table 3:** The main bioinformatics tools used to analyze the role of CDKN1A in GBM.

**Database**	**Samples**	**URL**	**References**
MalaCards	Tissues	https://www.malacards.org/	[49]
FunRich	–	http://www.funrich.org	[50]
GEPIA	Tissues	http://gepia.cancer-pku.cn/	[51]
GlioVis	Tissues	http://gliovis.bioinfo.cnio.es/	[52]
Wanderer	Tissues	http://maplab.imppc.org/wanderer/	[53]
THPA	Tissues	http://www.proteinatlas.org/	[54]
CCLE	Cell lines	https://portals.broadinstitute.org/ccle/	[55]
STRING	–	http://string-db.org/	[56]
Cytoscape	–	https://cytoscape.org/	[58]
DIVID	Tissues	https://david.ncifcrf.gov/	[60]
TISIDB	Tissues	http://cis.hku.hk/TISIDB	[61]

Gene expression profiling interactive analysis (GEPIA) and GlioVis were used to explore the relationships between the values of the four HTLV-1 infection-related genes and GBM prognoses. GEPIA, an interactive web server, can analyze RNA sequencing expression from TCGA and the GTEx projects [51]. GlioVis is a web-based tool and contains a large collection of brain tumor entries [52]. We then estimated the relevance among the CDKN1A expression and clinicopathological parameters of GBM patients using clinical data downloaded from Wanderer, which is an interactive viewer containing gene expression profiles from TCGA [53].

The expression levels of CDKN1A in GBM tissues and cell lines were analyzed in the following four databases. The GEPIA and GlioVis databases were also used to analyze the mRNA expression levels of CDKN1A in GBM tissues. The Human Protein Atlas (HPA) was used to analyze the protein expression levels of CDKN1A in GBM tissues [54]. An encyclopedia of cancer cell lines (CCLE) [55] was used to analyze CDKN1A expression in different GBM cell lines. Chemotherapy-related datasets, including GSE43452, GSE65363 and GSE80729, were used to explore the impact of CDKN1A expression on the chemotherapy response of GBM.

Next, CDKN1A-associated coexpression genes in GBM pathology were downloaded from GlioVis. We constructed a protein-protein interaction (PPI) of these coexpression genes from STRING which is a database that integrates known and predicted PPI networks from many organisms [56, 57]. Then, Cytoscape software, a tool that visually integrates networks with phenotypes and gene expression profiles was used to perform detailed visualization [58, 59]. Moreover, we utilized the database for annotation, visualization and integrated discovery (DAVID) to conduct a KEGG pathway analysis of the coexpression genes with CDKN1A in GBM [60].

The TISIDB database is an integrated repository portal for tumor-immune system interactions [61]. In this study, using the TISIDB database, we analyzed the correlation between CDKN1A expression in GBM patients and tumor-infiltrating lymphocytes (TILs) and immunomodulators.

### Cells and reagents

The TMZ-resistant glioma cell lines (U87-R and T98G-R) and their parental cell lines (U87 and T98G) were established and cultured as previously described [20]. MK2206 was purchased from Selleck Chemicals and dissolved in dimethylsulfoxide (DMSO) (Sigma, USA). The exposed concentrations of TMZ, MK2206 were 200 and 5mM, respectively.

### Colony formation assay

For the colony formation assay, the methods were described previously [62].

### Protein extraction and quantification

Protein extraction was carried out as previously described [63]. Protein concentration was determined by DC (detergent compatible) protein assay (Bio-Rad Laboratories, USA) according to manufacturer’s instructions.

### Western blot

Protein samples were resolved by SDS-PAGE, transferred to polyvinylidene difluoride membrane, and hybridized with antibodies specific to CDKN1A (2946; Cell Signaling Technology), p-CDKN1A (PA5-37519; Invitrogen, USA), AKT (9272s, Cell Signaling Technology, USA), p-AKT (4060, Cell Signaling Technology, USA), GAPDH (sc-47724; Santa Cruz, USA). The blots were developed by the enhanced chemiluminescence reagent (Thermo Scientific Pierce ECL, USA), visualization of the protein bands was conducted in the ChemiDoc XRS system (Bio-Rad, USA).

### Transient transfection

For transient transfections, the two TMZ-resistant glioma cell lines (U87-R and T98G-R) were transfected with siRNAs for CDKN1A (siCDKN1A, 5, - GAUGUCCGUCAGAACCCAUGCGGCA-3’) using Lipofectamine 3000 reagent (Invitrogen, USA) according to the manufacturer's instruction. After the indicated incubation times, the cells were harvested and analyzed.

### Statistical analyses

Statistical analyses were performed with SPSS 12.0 software (IBM Analytics, USA). All experiments were performed in at least triplicate with mean ± SD subjected to Student’s t-test. Kaplan-Meier analysis was performed to analyze survival rates for GBM. The differential mRNA expression between cancer and noncancer tissues or between the control group and treatment group were analyzed using Student’s t-test. The associations between CDKN1A expression and clinicopathologic characteristics in GBM patients were assessed using the Kruskal–Wallis rank test or Mann–Whitney U test. Correlations between genes were analyzed using Pearson's correlation coefficient. *p < 0.05, and **p < 0.01 were defined as statistically significant.

### Ethical statement

The authors are accountable for all aspects of the work in ensuring that questions related to the accuracy or integrity of any part of the work are appropriately investigated and resolved. None of the data have been previously published or appeared in copyrighted form elsewhere, and not previously published or unpublished data were cited in this paper. No ethics approval was required for this bioinformatics article, as it did not involve patients or patient data.

## Supplementary Material

Supplementary Figure 1

Supplementary Tables 1, 2 and 3

Supplementary Table 4
