# Supplementary material for: The novel roles of virus infection-associated gene CDKN1A in chemoresistance and immune infiltration of glioblastoma
Source: Aging (Albany NY). 2021 Feb 17;13(5):6662–80. doi: 10.18632/aging.202519 (PMC7993694; doi:10.18632/aging.202519)
Supplement: Supplementary Figure 1 [file aging-13-202519-s001.pdf]

SUPPLEMENTARY FIGURE

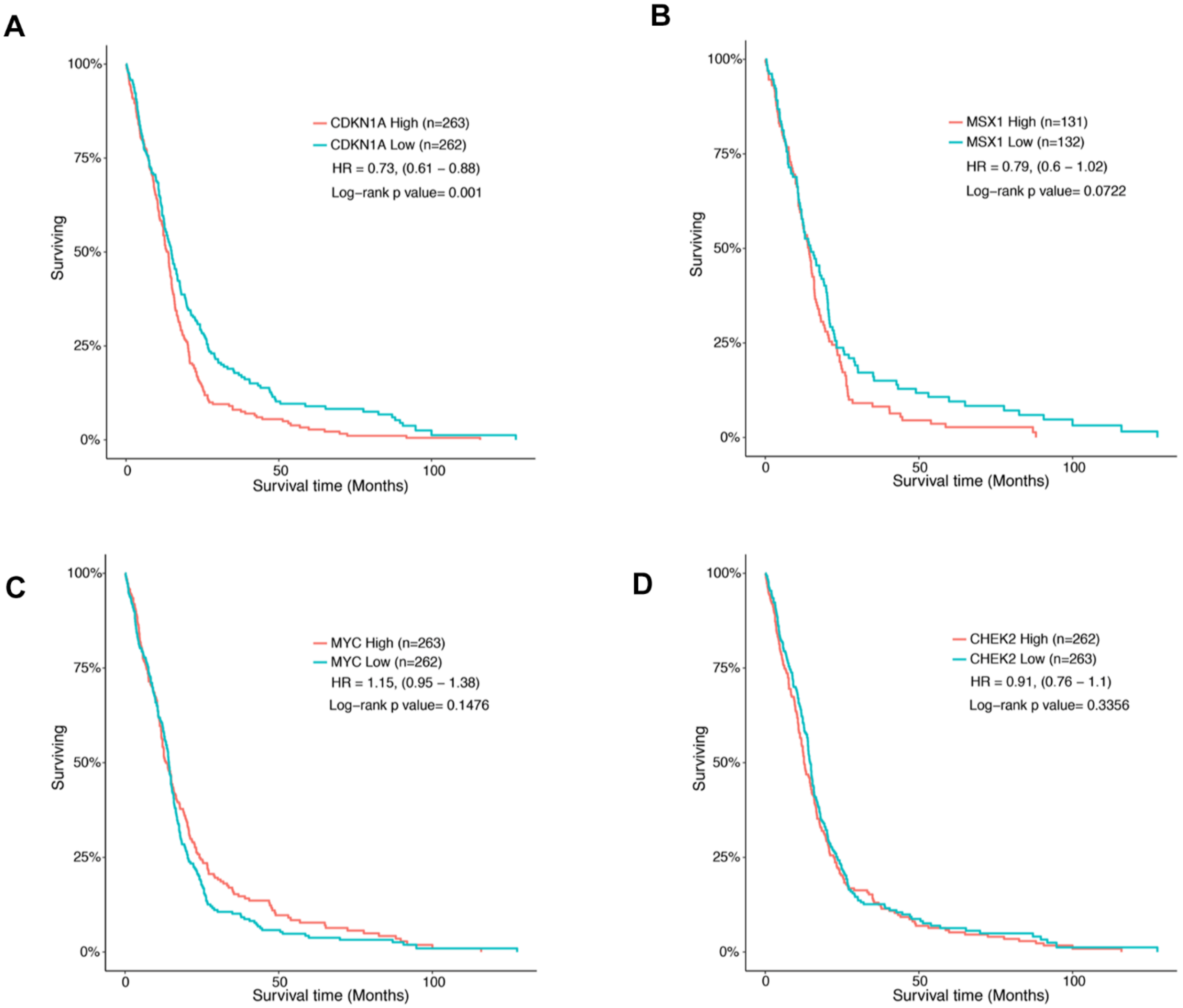

Supplementary Figure 1. Prognostic values of CDKN1A, MSX1, MYC and CHEK2 in GBM. (**A–D**) Kaplan-Meier analysis of overall survival among the samples with high expression levels of the four genes and those with low expression levels in GBM by using GlioVis database.
