# Supplementary material for: The novel roles of virus infection-associated gene CDKN1A in chemoresistance and immune infiltration of glioblastoma
Source: Aging (Albany NY). 2021 Feb 17;13(5):6662–80. doi: 10.18632/aging.202519 (PMC7993694; doi:10.18632/aging.202519)
Supplement: Supplementary Tables 1, 2 and 3 [file aging-13-202519-s002.pdf]

SUPPLEMENTARY TABLES

Supplementary Table 1. The 74 co-differentially expressed genes (co-DEGs) in all three GEO datasets.

GDF15	HSPA4L	CYP26B1	DOCK7	IRS1	RRM2B	CHEK2	BOLA2	GAB1	SLC25A45
CDKN1A	ACTA2	AEN	TRIM22	CYFIP2	ERCC6L	CA9	CSF3	NTPCR	CDK5RAP2
BTG2	PLXNB2	SNAI2	SYNCRIP	TNFRSF10B	SPATA18	TMEFF2	SERPINE1	BNIP3	DUSP5
HIST1H2BK	STOM	FST	UNG	CYP1B1	RBM4	NRG1	MYLK	RALGDS	GAS6
SESN1	SUGCT	KIF2C	DDIT4	TIGAR	MYC	COL12A1	PLOD2	C10orf88	ARPP21
DDB2	IP6K2	ZMAT3	CDH4	TIMELESS	MCL1	ZNF337	TNFRSF14	GPER1	SGK223
TP53INP1	XPC	SOCS3	SDC1	FAM111A	CMBL	CBLL1	MSMO1	PTPRR	NHS
CBX4	AHNAK	MSX1	PARVB						

Supplementary Table 2. The Genes in the Human T-cell leukemia virus 1 infection.

AKT3	CDK2	CDK4	CDKN1A	CDKN2A	CDKN2B	CDKN2C	ANAPC10	CREB3	KAT5
ADCY2	ADCY3	ADCY5	CHEK1	ADCY6	CHEK2	ADCY7	ADCY8	CHUK	ADCY9
ATF2	CREBBP	ATF6B	CSF2	CREB3L4	DLG1	E2F1	E2F2	E2F3	EGR1
CRTC2	ELK1	ELK4	EP300	AKT1	AKT2	ETS1	ETS2	FDPS	CRTC1
KAT2A	GPS2	SLC25A4	SLC25A5	SLC25A6	ANAPC2	ANAPC4	HLA-A	HLA-B	HLA-C
HLA-DOA	HLA-DOB	HLA-DPA1	HLA-DPB1	HLA-DQA1	HLA-DQA2	HLA-DQB1	HLA-DRA	HLA-DRB1	HLA-DRB3
HLA-E	HLA-F	HLA-G	HRAS	XIAP	ICAM1	IKBKB	IL1R1	IL2	IL2RA
IL6	IL15	IL15RA	ITGAL	ITGB2	JAK1	JAK3	JUN	KRAS	TBPL2
LTBR	MAD2L1	SMAD2	SMAD3	SMAD4	MAP3K1	MAP3K3	MMP7	MSX1	MSX2
ATM	NFATC1	NFATC2	NFATC3	NFATC4	NFKB1	NFKB2	NFKBIA	NFYB	NRAS
ANAPC11	PIK3CA	PIK3CB	PIK3CD	PIK3R1	PIK3R2	POLB	ATR	PPP3CA	PPP3CB
PPP3R2	PRKACA	PRKACB	PRKACG	VAC14	MAPK1	MAPK3	MAPK8	MAPK9	MAPK10
B2M	PTEN	BAX	RAN	RANBP1	RB1	CCND1	RELA	RELB	BCL2L1
CREB3L2	CRTC3	SLC2A1	SPI1	SRF	STAT5A	STAT5B	TBP	TCF3	BUB1B
TGFB2	TGFB3	TGFBR1	TGFBR2	TSPO	TLN1	TNF	TNFRSF1A	TP53	VDAC1
XPO1	ZFP36	IL1R2	FOSL1	CALR	CANX	TRRAP	SLC25A31	TLN2	MAD1L1
PIK3R3	IKBKG	CDC23	NRP1	KAT2B	CDC16	CCNA2	CCNA1	CCND2	CCND3
CREB3L1	CCNB2	CCNE2	CD3D	CD3E	CD3G	BUB3	CD4	PTTG1	TBPL1
ESPL1	CDC20	CDC27	MAP2K2	ANAPC1	TGFB1	VDAC3	RANBP3	MAP3K14	CREB5
ADCY1	TNFRSF13C	EGR2	FOS	HLA-DMA	HLA-DRB4	IL2RB	LCK	MYC	ANAPC5
PPP3CC	MAP2K1	MAP2K4	TERT	VDAC2	CREB3L3	CCNE1	CD40	PPP3R1	ANAPC7
PTTG2	CREB1	ADCY4	CDC26	HLA-DMB	HLA-DRB5	IL2RG	LTA	ATF4	

Supplementary Table 3. The 99 differentially expressed genes that interact with CDKN1A from GlioVis database.

CHI3L1	SERPINE1	TIMP1	IL8	CHL1	EFEMP2	ANXA2	HRH1	LOXL1	INA
PTX3	FCGBP	TNFAIP6	ABCC3	DDB2	SOD2	FCGR2B	S100A10	C5AR1	SH3GL2
LTF	EMP3	C21orf62	IGFBP2	SERPINA1	ANXA1	ARSJ	PIPOX	EMP1	FGF13
CDKN1A	CA12	DPYD	NAMPT	STEAP3	NR2E1	MAOB	PTRF	ANXA2P2	FERMT1
CXCL14	NNMT	MOXD1	DIRAS3	CD44	TREM1	FZD7	S100A8	PLTP	SNAP91
CHI3L2	PLA2G2A	OXTR	CLEC5A	TAGLN	LIF	PYGL	WWTR1	KIAA0226L	CD24
PLA2G5	F13A1	SERPINA3	EFEMP1	HAMP	SCG2	CSTA	FAM129A	VSIG4	DCX
PDPN	ADM	SRPX	SERPING1	ANGPTL4	C1S	RNASE2	COL5A1	PLAU	LPPR1
CCL2	CD163	C1R	IGFBP3	POSTN	PLAUR	C1RL	FABP7	CA10	TOX3
LOX	LGALS3	CFI	TRIM22	ACSS3	ALOX5AP	TGFBI	UPP1	DLL3	
